# Nurse-led self-care interventions for older adults with multiple chronic conditions: A protocol for a systematic review and network meta-analysis

**DOI:** 10.1371/journal.pone.0298082

**Published:** 2024-01-30

**Authors:** Heejung Choi, GyeongAe Seomun

**Affiliations:** 1 College of Nursing, Korea University, Seoul, Republic of Korea; 2 Transdisciplinary Major in Learning Health Systems, Graduate School, Korea University, Seoul, Republic of Korea; University of Benghazi, LIBYA

## Abstract

The importance of self-care interventions is progressively recognized, marked by a rapidly aging population that results in growing demands on costly tertiary and institutional care services, placing substantial pressure on health and social care systems. Against this background, in this study, we will provide evidence for the impact of self-care interventions on health-related quality of life by focusing on interventions led by nurses who can employ integrated approaches. Several databases, including Ovid MEDLINE, Cochrane, CINAHL, Embase, and PubMed, will be searched along with gray literature to prevent biased results. There will be no time restrictions, and all literature with English abstracts will be included. Using the Template for Intervention Description and Replication framework, we will evaluate intervention characteristics. The primary outcome will be health-related quality of life, and the secondary outcomes will be symptom burden, physical function, and cost-effectiveness. Pairwise and network meta-analyses will be performed, and network geometry and the surface under the cumulative ranking curve will be used to determine which type of nurse-led self-care intervention is best for health-related quality of life for older adults with multiple chronic conditions. We will secure study quality through PRISMA, AMSTAR 2, RoB 2.0, and the GRADE checklist. To ensure the quality of network meta-analysis, similarity will be checked based on the PICO framework. The consistency of the network meta-analysis results will be checked to ensure transitivity by using the Bayesian hierarchical framework. The study protocol is registered with PROSPERO (CRD42022365467).

## Introduction

As the aging population grows, there is an increasing number of older adults with multiple chronic conditions, defined as health conditions characterized by the simultaneous existence of two or more chronic diseases [1–3]. For older adults with multiple chronic conditions, the patient care approach must differ from that of a single chronic disease [1]. This is because focusing on a single disease can lead to problems such as polypharmacy, conflicting medical recommendations, adverse drug reactions, and drug-drug interactions [4]. Such an approach also contributes to fragmented and unintegrated healthcare services, placing a heavy burden on patients with multiple chronic conditions [1]. As a result, patients with multiple chronic conditions often experience confusion regarding their diseases, how to manage them, and how to communicate with healthcare providers [5]. They also expect a single professional to take responsibility for their care and guide treatment decisions [1].

Self-care is an alternative patient care approach for individuals with multiple chronic conditions, as outlined in current guidelines from the US and UK governments. The US Department of Health and Human Services (DHHS) advocates for transforming patient care for multiple chronic conditions with four key strategic goals: promoting proven self-care management in individuals, strengthening healthcare systems, providing better information and tools, and facilitating research on effective interventions [6]. In the UK, the National Institute for Health and Clinical Excellence (NICE) has provided guidelines emphasizing personalized care, understanding patients as individuals, active patient participation in care, and the importance of continuity of care and relationships [7]. Both the DHHS and NICE highlight the significance of self-care as a strategic approach to addressing multiple chronic conditions.

According to the World Health Organization (WHO), self-care refers to individuals, families, and communities promoting health, preventing disease, staying healthy, and managing illnesses without relying solely on healthcare professionals [8,9]. The middle-range theory of self-care in chronic illness also clearly defines self-care as maintaining health through practices that promote well-being and effectively manage the illness [10]. Self-care is crucial for potential benefits like enhancing health outcomes, reducing hospitalization and healthcare expenditure [11–13], and fulfilling the preference of most older adults to age in their familiar places, thereby increasing satisfaction, sense of control, and quality of life [13–16]. Conversely, the decline in independence among older adults leads to a diminished sense of self-confidence, achievement, identity, and life meaning, indicating a decrease in overall quality of life and life satisfaction [15].

Self-care interventions are tools that facilitate self-care [8]. They are multifaceted, encompassing various elements such as educational sessions to enhance patient behaviors and promote independent symptom monitoring, psychosocial tactics, goal setting, motivational interviewing, and developing problem-solving and decision-making skills [11,17]. These interventions exhibit variability in formats (individual or group), settings (hospital or home), delivery methods (face-to-face or telephone), lead coordinators (such as nurses), and degree of the intervention [11,18]. Additionally, self-care interventions for older adults typically concentrate on instructing individuals on managing age-related challenges encountered in their daily lives [15]. These interventions have demonstrated positive outcomes for the physical health of older adults, including reduced limitations in daily activities and health-related distress [15,19,20].

Nurses have a unique role, distinguishing them from other healthcare professionals in promoting self-care practices among older adults [21,22]. Various research supports their proficiency in preventive measures, which consists of them utilizing thorough assessments for early detection of health concerns [21,22], embracing a holistic approach to care for multiple health complaints [22,23], making referrals to other healthcare professionals in a multidisciplinary setting when necessary [22,24], and establishing trusting relationships with older adults [22,25]. Therefore, nurses are increasingly proposed as lead coordinators in interdisciplinary case management [24,26–28].

Although a clear and consistent definition of nurse-led intervention has yet to be established, such interventions primarily feature the participation of nurses while also allowing for involvement from other relevant healthcare professionals [11,25]. Nevertheless, independence in practice and decision-making is recognized as a fundamental aspect of nurse-led interventions [25,29,30]. Nurses have various roles in nurse-led interventions, including coordinators, case managers, consultants, educators, navigators, practitioners, and researchers [24,31–33]. Current evidence supports the positive impact of nurse-led interventions on managing chronic diseases, including systolic blood pressure, pain control, and health services utilization [11,34,35].

In the definition of nurse-led self-care interventions, the terms “nurse-led” and “self-care” appear contradictory. Though patients take responsibility for self-care, nurses play a crucial role in collaborating with patients and teams to support patients in independent self-care, using their autonomy as team leaders [11,36]. The mechanism involves focusing on specific clinical characteristics of patients with chronic diseases, creating personalized plans, and enhancing disease knowledge through diverse health education and interdisciplinary communication [4,36,37]. In nurse-led self-care interventions, teaching patients problem-solving complements traditional health education, which provides disease-specific knowledge and skills [38,39]. This approach enhances patients’ independence and ultimately improves their quality of life [38,39]. Additionally, nurses address negative emotions during the interventions and promote medical adherence, providing integrated care [36]. Previous systematic reviews have shown that nurse-led self-care interventions for older adults with a single chronic disease can promote health-related quality of life (HRQOL) [40], self-efficacy, and self-care behavior and reduce depression levels [11] and mortality [40]. However, despite many older adults confronting multiple chronic conditions instead of a single chronic disease [1–3], to our knowledge, no systematic review examines the impacts of nurse-led self-care interventions for older adults with multiple chronic conditions.

Regarding adequate outcomes of self-care interventions for patients with multiple chronic conditions, the NICE has emphasized the need to evaluate quality of life, clinical outcomes, and cost-effectiveness [7]. Additionally, Cochrane reviews in 2012 and 2016 [41], as well as NICE [7], highlighted the need to focus on outcomes that remain relevant across diseases, such as quality of life, symptom burden (polypharmacy, multiple appointments), physical function, and cost-effectiveness [1]. Therefore, we selected HRQOL as the primary outcome because specific outcomes focused on each disease cannot reflect complex conditions. Moreover, the eventual goal of self-care interventions is HRQOL improvement rather than disease control. However, recent systematic reviews have reported conflicting results, and doubts remain regarding whether self-care intervention can affect HRQOL [41–43].

Considering the many confounding factors in the context of multiple chronic conditions, we will conduct this study with clear boundaries. The target population will be limited to individuals aged 65 or above. We will only consider nurse-led interventions for the 16 types of self-care presented by the WHO. Multiple chronic conditions will be defined as the simultaneous presence of two or more chronic conditions out of the 20 presented by the Office of the Assistant Secretary for Health (OASH). Additionally, intervention characteristics, number of chronic conditions, types of chronic conditions, gender, patient care experiences, and specific patient health behaviors (e.g., exercise) will be analyzed using the Template for Intervention Description and Replication (TIDieR) checklist to make a structured evaluation.

Network meta-analysis (NMA) is a novel analytical method that enables direct and indirect comparisons and allows researchers to collect evidence to concurrently include various pairwise comparisons across various interventions to guide decision-making in clinical settings [44,45]. The advantages of NMA are that it can be used to rank self-care interventions for individuals with multiple chronic conditions and then offer evidence-based data to assist in treatment decisions [45]. Therefore, in this study, the results of the NMA will help select an optimal nurse-led self-care intervention, either on its own or in combination with other interventions, to manage patients with multiple chronic conditions.

### Primary aim

We will conduct a systematic review to evaluate the effects of nurse-led self-care interventions for older adults with multiple chronic conditions. This review aims to identify the most effective intervention for HRQOL. Both pairwise meta-analysis and NMA will be conducted.

### Review questions

#### Primary review question

In older adults with multiple chronic conditions, what is the impact of nurse-led self-care interventions on HRQOL compared to waitlist, usual care controls, placebo, and other comparison groups?

#### Secondary review question

What is the ranking of nurse-led self-care interventions for improving HRQOL in older adults with multiple chronic conditions?

## Materials and methods

This systematic review and NMA will be performed according to the Preferred Reporting Items for Systematic Reviews and Meta-Analyses (PRISMA) extension statement for NMA [46]. The protocol is registered in the International Prospective Register of Systematic Reviews with registration number CRD42022365467 and is reported according to the guidelines provided in the PRISMA Protocols statement [47] (S1 Checklist).

### Criteria for study selection

A review will be performed to identify studies published from inception through January 26, 2023, along with gray literature searches, to prevent biased results. The PICOS format will be used to set the search strategy. To minimize language bias, English abstracts will be included in the search without language restrictions on full-text versions [48]. After completing the abstract review, full-text articles written in languages other than English will be evaluated using software tools or translations by language experts to maintain the clarity of the original content.

#### P (Population)

The study population will include older adults (≥ 65 years old) with multiple chronic conditions who have two or more chronic diseases out of the 20 chronic conditions presented by the OASH [49] as follows: diabetes, hypertension, cardiac arrhythmias, hyperlipidemia, stroke, asthma, congestive heart failure, chronic obstructive pulmonary disease, coronary artery disease, cancer, chronic kidney disease, arthritis, depression, schizophrenia, autism spectrum disorder, hepatitis, human immunodeficiency virus infection, osteoporosis, dementia, and substance abuse disorders.

#### I (Intervention)

This will include studies on nurse-led self-care interventions.

To identify studies that implement self-care interventions aligned with the components of self-care as outlined by the WHO, we aim to incorporate literature that encompasses the WHO’s concept of self-care into this study: self-management, self-awareness, and self-testing. Self-management comprises six sub-concepts: self-treatment, self-medication, self-examination, self-administration, self-injection, and self-use. Self-awareness comprises five sub-concepts: self-education, self-determination, self-regulation, self-efficacy, and self-help. Self-testing comprises five sub-concepts: self-screening, self-sampling, self-monitoring, self-diagnosis, and self-collection [8].

Additionally, we have referred to Riegel’s operational definition of self-care intervention and established the following selection and exclusion criteria [17]. The inclusion criteria for self-care interventions are as follows: (1) enhancing patient behaviors, (2) promoting independent symptom monitoring, and (3) fostering problem-solving and decision-making skills. The exclusion criteria are as follows: (1) interventions that only target a single health behavior as they lack the holistic approach needed for managing chronic illnesses, (2) interventions that involve patient-monitored symptoms without patient control or decision-making, thus not qualifying as self-care, (3) telemonitoring that involves provider-directed actions, and (4) interventions aimed at increasing self-care confidence or intention without actual behavior change.

We define nurse-led interventions as interventions characterized by nurses’ independence in their practice and decision-making, using the criteria outlined by McParland et al. [50]. Therefore, nurse-led interventions must fulfill at least one of the following conditions: (1) nurses oversee the service, (2) nurses undertake the responsibility of managing a specific caseload of patients, and (3) nurses demonstrate a discernible level of autonomy in their practice and decision-making compared to medically led care.

Finally, the criteria for selecting literature on nurse-led self-care intervention aim to identify documents that collectively satisfy the criteria mentioned for self-care, self-care intervention, and nurse-led intervention.

#### C (Control)

The control groups will include a waitlist, usual care controls, placebo, and various types of nurse-led self-care interventions as comparison groups. We will investigate possible clinical and statistical heterogeneity within the control groups (waitlist, usual care controls, placebo, comparison groups) to determine if they can be treated as a single node for analysis.

#### O (Outcome)

As previous studies recommended adequate interventions outcomes for patients with multiple chronic conditions, the primary outcome will be HRQOL [1,7], as disease-specific outcomes cannot reflect complex disease conditions [7]. The eventual goal of nurse-led self-care interventions is HRQOL improvement rather than disease control. Additionally, symptom burden (polypharmacy, multiple appointments), physical function, and cost-effectiveness, determined using reliable instruments, will be included as secondary outcomes.

#### S (Study design)

This study will only involve randomized controlled trials. Reviews, letters, editorials, and protocols will be excluded.

### Data sources and search strategy

We will conduct an extensive literature search using the Ovid MEDLINE, Cochrane Central Register of Trials, CINAHL, Embase, and PubMed electronic databases, from inception through January 26, 2023. Controlled vocabulary (MeSH, Emtree, and CINAHL Subject Headings) and text will be adopted, mainly including “older adults” AND “multiple chronic conditions (MCC)” AND “nurse-led self-care intervention” AND “health-related quality of life (HRQOL)” AND “randomized controlled trial (RCT).” Pilot searches have been performed to determine the rate and accuracy of the retrieved literature. We will consult with experts in literature searches to identify the most appropriate search term strategy for our research topic. The publication date and status will not be limited. We will also screen the reference lists to obtain validated studies. Additionally, systematic searches of gray literature will be performed. The search strategy for Ovid MEDLINE can be found in the S1 File.

### Screening procedures for eligible studies

The initial search results will be imported into Covidence, a software tool used for systematic reviews (Veritas Health Innovation, Melbourne, Australia; available at www.covidence.org), which will automatically remove duplicates. Next, a single researcher will screen titles and abstracts. Another researcher will check a random selection of 10% of the screened studies to validate the included studies. Subsequently, two researchers will independently perform full-text reviews of the extracted data to select literature that meets all the inclusion criteria. Any discrepancies in the findings of the two researchers will be addressed and resolved by consulting a third researcher. The PRISMA flowchart will define the reasons for excluding studies.

### Assessment of risk of bias

The eligible randomized controlled trials will be appraised according to the Cochrane Risk of Bias Checklist for Randomized Trials 2.0 [51]. This tool comprises the following five bias domains for evaluation: (1) randomization process bias, (2) bias due to deviations from planned interventions, (3) bias due to missing outcome data, (4) outcome measurement bias, and (5) reported result extraction bias. The risk of bias assessment for each domain will be categorized as “low risk of bias,” “some concerns,” or “high risk of bias.” Each researcher will examine the risk of bias in each study. A third researcher will settle discrepancies or disagreements and make a final decision.

### Data extraction

Two researchers will individually extract data using a pre-planned form. General information (author, publication year, inclusion and exclusion criteria, outcomes) will be extracted from the included studies. The intervention effects will be assessed using results measured at baseline and post-intervention. To compare interventions between studies consistently, we will summarize a list of intervention characteristics that are essential determinants of effectiveness [52]. Intervention characteristics will be extracted using the TIDieR framework for a brief description and to illustrate why (theory, rationale), what (informational materials, procedures), who (intervention provider), how (modes of delivery), where (location), when and how much (number of sessions), tailoring (personalized, titrated, or adapted plan), modifications (reason for changes) and how well (attrition, compliance) [52]. Two researchers will independently extract data from all studies, and a third researcher will make a final decision in case of any discrepancies or disagreements. Intervention types will be classified according to the previously mentioned WHO categories. To eliminate errors and ensure the correctness of the data extracted by both researchers, the data will be compared until a consensus is reached.

### Data synthesis and analysis

To synthesize and analyze the selected studies, we will perform descriptive and quantitative statistics using R version 4.2.2 (R Foundation for Statistical Computing, Vienna, Austria). Narrative data will be synthesized using the TIDieR framework. General information will be presented, including settings, participants, intervention tools, duration, outcomes with measured tools, mean differences, standard deviations, sample size, and follow-up. Specifically, the key information will be outlined for nurse-led self-care interventions according to the TIDieR framework presented above [52].

In quantitative synthesis, direct meta-analyses will be performed to compare the efficacy of nurse-led self-care interventions for older adults with multiple chronic conditions using the inverse variance random‐effects model to combine outcomes with clinical or statistical heterogeneity [53]. We will use mean differences or standardized mean differences for continuous outcomes and odds ratios for binary outcomes, and 95% confidence intervals and two-sided p-values will be calculated for each outcome. If a study does not provide mean differences and standard deviations, corresponding values will be assumed and obtained according to the Cochrane Handbook. A pairwise meta-analysis with a random-effects model will be conducted when there are multiple randomized comparative clinical trials with multiple interventions. The purpose will be to assess the impact of various nurse-led self-care interventions on HRQOL outcome measures. The chi-squared test will be used to measure heterogeneity between studies [51]. Inconsistencies between studies will be assessed using I^2^ tests (I^2^ < 40% will be considered “not important,” and I^2^ > 50% will be considered “substantial”) [51]. Subgroup analyses will be performed on the primary outcome (HRQOL) to examine the effects of different sources owing to intervention characteristics, number of chronic conditions, types of chronic conditions, gender, patient care experience, and targeted patient health behaviors (e.g., exercise) to identify confounding factors other than nurse-led self-care interventions.

Second, we will conduct an NMA using meta-regression, a commonly used approach in many network studies [51]. The network diagram will be created using the R software. To ensure the quality of NMA, we will check the similarity through loop-specific inconsistency estimation, separate indirect from direct evidence, and incorporate design-by-treatment interaction models based on the PICO framework [51,54]. The consistency of the NMA results will be checked to ensure transitivity by using a Bayesian hierarchical framework [55]. Furthermore, we will utilize the surface under the cumulative ranking curves to evaluate the rankings of the nurse-led self-care interventions for HRQOL [56].

### Assessment of publication bias

We will employ a comparison-adjusted funnel plot to assess publication bias to detect potential small study effects within the NMA [51].

### Assessment of evidence quality

Based on the GRADE Working Group guidelines, two researchers will evaluate the quality of treatment effect estimates from the NMA, and a third researcher will resolve any discrepancies. The GRADE Working Group will guide the following approach: (1) providing both direct and indirect treatment estimates for every comparison in the evidence network, (2) assessing the quality of each estimate of direct and indirect effects, and (3) demonstrating and (4) evaluating the quality of each NMA effect estimate [57].

### Ethical considerations

This study has been granted an exemption by the Institutional Review Board for Ethical Approval at Korea University as it is a secondary literature review (exemption number: KUIRB-2022-0359-01).

### Validity and reliability

To ensure validity and reliability, this systematic review and NMA will rigorously adhere to the requirements of the PRISMA-NMA [58], Cochrane Handbook [51], PRISMA [59], and A MeaSurement Tool to Assess systematic Reviews 2 (AMSTAR 2) [60].

## Discussion

The need for self-care interventions is being emphasized by international organizations such as the DHHS, NICE, and WHO [6–8]. Self-care intervention is an integrative approach that focuses on the daily experience of patients. Among multidisciplinary teams providing self-care interventions for older adults with multiple chronic conditions, nurses are the healthcare professionals who can most easily apply this integrated approach in their interactions with patients. This study is significant because it aims to measure the impact of nurse-led self-care interventions on patients’ health improvement.

To do so, we will use the conceptual framework of self-care presented by the WHO, which has 16 subitems across three main concepts: self-management, self-awareness, and self-testing [8]. However, only some self-care types have been widely explored in previous studies. As all 16 types will be included in the search strategy in this study, the extracted self-care interventions will reflect the context of interventions linked to the health care system as the WHO intended.

To our knowledge, published systematic reviews have not drawn firm conclusions regarding interventions that improve HRQOL in patients with multiple chronic conditions [41,43,61]. This study will offer evidence for the impact of self-care intervention on HRQOL by focusing on nurse-led interventions and by using an alternative method such as NMA.

We acknowledge certain limitations in this study. First, although we aim to use the OASH list of 20 chronic diseases, the list has certain restrictions. It was developed using the US DHHS data system and excludes certain conditions found in patients with multiple chronic conditions, which limits its applicability to all types of chronic conditions. However, the benefit of the OASH list is that it uses the International Classification of Diseases codes, allowing for the identification and utilization of chronic conditions in different data systems [49]. This feature enables comparability with health data from other countries [49]. Additionally, if the OASH working group or global organizations like the WHO refine the chronic condition list based on country-specific prevalence rates, the value of the OASH list could increase. Moreover, despite excluding certain chronic conditions from the OASH list, using a standardized list of chronic conditions is important. This is because the variability in criteria for chronic condition classification resulting from various definitions used in studies can lead to a lack of comparability in defining multiple chronic conditions. Therefore, conducting studies that examine interventions for multiple chronic conditions based on the OASH list is meaningful as it provides clear criteria for categorizing chronic conditions and presenting research outcomes. To our knowledge, no systematic review has examined self-care intervention for all 20 chronic conditions in the OASH list. While not all chronic conditions are included, incorporating all 20 criteria of the list is still significant as it involves a substantial number of chronic conditions. Second, outcome measures may vary across studies. However, the outcomes will be presented using standardized mean differences and standardized values to address this heterogeneity issue. Finally, there is a risk that the outcomes in the selected studies may not be able to be combined into an NMA. Every systematic review and meta-analysis faces limitations regarding the number of available studies, the level of intervention details provided in the study reports, and the data types for effect size computation. We will make every effort to include all relevant studies and reach out to the authors of primary studies that lack adequate data for calculating effect sizes. If there is an insufficient number of available studies, we will supplement the analysis with observational data, including non-randomized studies, following Cochrane guidelines [51]. However, this study will still be meaningful as it will be the first systematic review of patients with multiple chronic conditions that utilizes NMA to compare intervention variability and characteristics of multiple chronic conditions.

Lastly, this study has implications for nursing practice. The findings will contribute to identifying the characteristics of nurse-led self-care interventions for older adults with multiple chronic conditions, determining effective intervention strategies, and providing a basis for the development of community and clinical practice guidelines for older adults with multiple chronic conditions. Furthermore, this study’s results can potentially improve the quality of life among older individuals with multiple chronic conditions. Therefore, this study seeks to examine the dynamics of nurse-led self-care intervention research for older adults with multiple chronic conditions while also synthesizing individual study outcomes to propose effective directions for nurse-led self-care interventions.

## Supporting information

S1 Checklist PRISMA-P 2015 checklist. (DOCX)

S1 File Search strategies and grey literature information source. (DOCX)
