# Supplementary material for: Nurse-led self-care interventions for older adults with multiple chronic conditions: A protocol for a systematic review and network meta-analysis
Source: PLoS One. 2024 Jan 30;19(1):e0298082. doi: 10.1371/journal.pone.0298082 (PMC10826940; doi:10.1371/journal.pone.0298082)
Supplement: S1 File — (DOCX) [file pone.0298082.s002.docx]

**S1 File. Search strategies and grey literature information sources**

**Search period: from inception to Jan 26 2023**

**I. Search strategies for Ovid Medline**

1. aged/ or aging/ or geriatrics/

2. (elder* or older people or older person or older adult* or older patient* or old people or old person or old patient* or old adult* or frail* or geriatric* or senior*).ti,ab,kw.

3. 1 or 2

4. multiple chronic conditions/ or comorbidity/ or chronic disease/

5. (comorbidit* or multimorbidit* or co morbidit* or multi morbidit* or multiple condition* or multiple diseas* or multiple chronic conditions or multiple chronic health conditions or multiple chronic medical conditions or multiple chronic illnesses or chronic illness* or chronic condition* or chronically ill).ti,ab,kw.

6. 4 or 5

7. hypertension/ or essential hypertension/ or hypertension, malignant/

8. (hyperten* or hypertension or hypertensive or high blood pressure or high blood pressures or systolic blood pressure or diastolic blood pressure or raised blood pressure or primary hypertension or primary hypertension or essential hypertension).ti,ab,kw.

9. 7 or 8

10. heart failure/ or Heart Failure, Diastolic/ or Heart Failure, Systolic/

11. (heart failure or CHF or congestive heart failure or HF or cardiac failure or heart decompensation or right sided heart failure or myocardial failure or congestive heart failure or left sided heart failure).ti,ab,kw.

12. 10 or 11

13. coronary artery disease/

14. (coronary artery disease or coronary arteriosclerosis or coronary atherosclerosis or angina pectoris or CAD or heart disease or myocardial infarction or unstable angina or angor pectoris or coronary thrombosis or acute coronary syndrome or myocardial ischemia or myocardial ischaemia or left main coronary artery disease or left main disease* or left main coronary disease).ti,ab,kw.

15. 13 or 14

16. arrhythmias, cardiac/

17. (cardiac dysrhythmia or cardiac arrhythmia* or arrhythmia or atrial arrhythm* or ventricular arrhythm*).ti,ab,kw.

18. 16 or 17

19. hyperlipidemias/ or dyslipidemias/ or hypercholesterolemia/ or hyperlipidemia, familial combined/ or hyperlipoproteinemias/ or hypertriglyceridemia/

20. (cholesterol or triglycerides or lipid* or hyperlipemia* or lipidemia* or lipemia* or dyslipidemia or dyslipoproteinemia* or hypercholesterolemias or high cholesterol level or elevated cholesterol* or hypercholesteremia* or combined hyperlipidemia or familial combined hyperlipidemia or multiple lipoprotein type or lipoprotein type hyperlipidemia or multiple lipoprotein type hyperlipidemia or hyperlipoproteinemia* or hypertriglyceridemia*).ti,ab,kw.

21. 19 or 20

22. stroke/ or cerebrovascular trauma/ or hemiplegia/ or paresis/

23. (stroke or strokes or hemipleg* or paresis or cerebrovascular trauma or cerebrovascular accident* or CVA or cerebrovascular apoplexy or brain vascular accident* or cerebrovascular stroke* or apoplexy or cerebral stroke* or acute stroke* or acute cerebrovascular accident* or cerebrovascular trauma or brain vascular trauma or brain vascular injur* or hemiplegia* or pares*).ti,ab,kw.

24. 22 or 23

25. arthritis/

26. (arthritis or rheuma or arthritides or polyarthritis or polyarthritides or oligoarthritis or oligoarthritides).ti,ab,kw.

27. 25 or 26

28. asthma/

29. (asthma* or status asthmaticus or bronchial hyper reactivity or wheez or bronchial or obstructive lung disease or bronchial Asthma).ti,ab,kw.

30. 28 or 29

31. autism spectrum disorder/

32. (autis* or autism spectrum disorder* or ASD or autism spectrum condition or autistic spectrum disorder* or Kanner's syndrome or Kanner Syndrome or Kanners Syndrome).ti,ab,kw.

33. 31 or 32

34. neoplasms/

35. (tumor or neoplasm or tumors or neoplasia or neoplasias or cancer or cancers or malignant or malignancy or malignancies or neoplasms or tumour or tumours or carcinoma or carcinomas).ti,ab,kw.

36. 34 or 35

37. renal insufficiency, chronic/

38. (chronic renal insufficiency or chronic kidney failure or chronic renal failure or chronic renal disease or chronic renal insufficienc* or chronic kidney insufficiency or chronic kidney disease* or chronic renal disease* or chronic kidney disorder* or CKD or ESRD or CRD).ti,ab,kw.

39. 37 or 38

40. pulmonary disease, chronic obstructive/

41. (chronic obstructive pulmonary disease* or chronic obstructive lung disease* or chronic bronchitis or COPD or COAD or chronic obstructive airway disease* or chronic airflow obstruction or chronic obstructive lung disease* or emphysema or chronic airflow obstruction*).ti,ab,kw.

42. 40 or 41

43. dementia/ or vascular dementia/ or Parkinson disease/ or secondary Parkinson disease/

44. (senile dementias or dementia* or amentia* or Alzheimer or Parkinson* or senile paranoid dementia* or senile paranoid or familial dementia* or Alzheimer Dementia* or Alzheimer's Disease or senile dementia or Alzheimer type dementia or ATD or Alzheimer type senile dementia or primary senile degenerative dementia or Alzheimer sclerosis or Alzheimer syndrome or Alzheimer's diseases or Alzheimer diseases or Alzheimers diseases or acute confusional senile dementia or presenile dementia or late onset Alzheimer disease or focal onset Alzheimer's disease or familial Alzheimer disease or FAD or familial Alzheimer diseases or early onset Alzheimer disease or presenile Alzheimer dementia or idiopathic Parkinson's disease or lewy body Parkinson's disease or Parkinson's disease or idiopathic Parkinson disease or lewy body Parkinson disease or primary Parkinsonism or paralysis agitans or secondary parkinson disease or symptomatic parkinson disease or symptomatic parkinsonism or secondary parkinsonism or secondary vascular parkinson disease or atherosclerotic parkinsonism).ti,ab,kw.

45. 43 or 44

46. depression/ or depressive disorder/ or dysthymic disorder/

47. (depression or depressive symptom* or emotional depression or depressive disorder* or depressive neuros* or endogenous depression* or depressive syndrome* or neurotic depression* or melancholia* or unipolar depression* or dysthymic disorders or dysthymia or persistent depressive disorder or dysthymia and chronic depression or major depressive disorder* or involutional paraphrenia or involutional paraphrenias or involutional psychos* or involutional psychoses or involutional depression or involutional melancholia).ti,ab,kw.

48. 46 or 47

49. diabetes mellitus/ or insulin resistance/

50. (diabetes mellitus or diabetes insipidus or diabetic or prediabetic state or scleredema adultorum or glucose intolerance or gastroparesis or insulin resistance or insulin sensitivity or noninsulin dependent diabetes mellitus or ketosis resistant diabetes mellitus or stable diabetes mellitus or type II diabetes mellitus or diabetes mellitus type 2 or DMII or DM2 or IDDM or NIDDM or maturity onset diabetes mellitus or MODY or slow onset diabetes mellitus or type 2 diabetes mellitus or maturity onset diabetes or type 2 diabetes or adult onset diabetes mellitus or noninsulin dependent or impaired glucose tolerance or impaired glucose tolerant).ti,ab,kw.

51. 49 or 50

52. hepatitis, chronic/

53. (hepatitis or chronic hepatitis or cryptogenic chronic hepatitis or chronic active hepatitis or chronic persistent hepatitides or chronic persistent hepatitis or chronic hepatitis B virus infection or chronic hepatitis B or chronic hepatitis C or chronic hepatitis D or chronic delta hepatitis or chronic delta hepatitides or autoimmune hepatitides or autoimmune chronic hepatitis or autoimmune chronic hepatitides or autoimmune hepatitis).ti,ab,kw.

54. 52 or 53

55. HIV/

56. (human immunodeficiency virus or human immunodeficiency viruses or human t cell lymphotropic virus type III or human t cell lymphotropic virus type III or human t cell leukemia virus type III or human t cell leukemia virus type III or LAV HTLV III or lymphadenopathy associated virus or lymphadenopathy associated virus or lymphadenopathy associated viruses or human t lymphotropic virus type III or human t lymphotropic virus type III or AIDS virus* or acquired immune deficiency syndrome virus or acquired immunodeficiency syndrome virus or HTLV III).ti,ab,kw.

57. 55 or 56

58. osteoporosis/

59. (osteoporos* or post traumatic osteoporos* or senile osteoporos* or involutional osteoporosis or age related osteoporos* or age related bone loss or age related osteoporos*).ti,ab,kw.

60. 58 or 59

61. schizophrenia/

62. (schizophrenia or schizophrenic disorder* or dementia praecox or catatonic schizophrenia* or disorganized schizophrenia* or hebephrenic schizophrenia* or paranoid schizophrenia* or delusional disorder* or treatment resistant schizophrenia or refractory schizophrenia or shared psychotic disorder* or shared paranoid disorder* or folie a deux or folie a trois).ti,ab,kw.

63. 61 or 62

64. substance-related disorders/

65. (substance misuse or substance related disorder* or (drug adj3 disorder*) or substance induced or organic mental disorder* or substance abuse* or substance dependenc* or substance addiction or chemical dependence* or drug dependence or drug addiction or prescription drug abuse or substance use* or drug abuse* or drug habituation or (substance adj3 disorder*)).ti,ab,kw.

66. 64 or 65

67. 6 or 9 or 12 or 15 or 18 or 21 or 24 or 27 or 30 or 33 or 36 or 39 or 42 or 45 or 48 or 51 or 54 or 57 or 60 or 63 or 66

68. 3 and 67

69. Nursing/ or Nursing Care/ or Nurses/ or Nurse-Patient Relations/ or Models, Nursing/ or Patient Care Team/ or Delivery of Health Care, Integrated / or Patient-Centered Care /

70. (nursing care* or nurse* or nursing intervention* or nurse patient relation* or nursing follow up* or non pharmacological intervention* or behavioral intervention* or interdisciplinary or nurse pharmacist* or nurse GP* or nurs*).ti,ab,kw.

71. 69 or 70

72. self care/

73. (self care* or self caring).ti,ab,kw.

74. 72 or 73

75. self-management/

76. (self manag* or self managable or self managament or self manage or self manageable or self managed or self manager or self managerial or self managers or self manages or self managing).ti,ab,kw.

77. 75 or 76

78. self medication/

79. (self medication* or non prescription or OTC drug or over the counter drug or self prescription).ti,ab,kw.

80. 78 or 79

81. (self treatment or self treated or self treating or self treat* or emergency treatment or SBET or standby emergency self treatment).ti,ab,kw.

82. self-examination/

83. (self examination* or self exam* or self surveillance).ti,ab,kw.

84. 82 or 83

85. (self injection or self inject* or self injected or self injectable).ti,ab,kw.

86. self Administration/

87. (self administration* or self administer* or self-administering medication).ti,ab,kw.

88. 86 or 87

89. (self-use or self-using or self-used).ti,ab,kw.

90. 77 or 80 or 81 or 84 or 85 or 88 or 89

91. self-testing/

92. (self testing or self tested or self test or rapid diagnostic test or RDT or home testing or home test or home based test or home based testing).ti,ab,kw.

93. 91 or 92

94. (self sampling or self sample or self samples or self sampled).ti,ab,kw.

95. (self screen* or self screening or self screened).ti,ab,kw.

96. diagnostic self evaluation/

97. (self diagnosis or self diagnosis or self diagnosed or self* diagnos*).ti,ab,kw.

98. 96 or 97

99. (self-collect* or self-collection or self collection or self collected or self collecting or self versus provider collected or self versus physician collected).ti,ab,kw.

100. (self-monitor* self-monitorable or self-monitored or self-monitoring or self-monitors).ti,ab,kw.

101. 93 or 94 or 95 or 98 or 99 or 100

102. awareness/ or consciousness/ or metacognition/ or self-perception/ or self concept/

103. (self awareness or self aware or diagnostic self evaluation or awareness or consciousness or metacognition or self perception or self concept or self appraisal or self conscious*).ti,ab,kw.

104. 102 or 103

105. (self help or self helping or self helped).ti,ab,kw.

106. health education/ or patient education as topic/ or behavior therapy/

107. (self-education or self education or health education or patient education or behavior therapy).ti,ab,kw.

108. 106 or 107

109. self-control/

110. (self regulation or self control or self regulat*).ti,ab,kw.

111. 109 or 110

112. self efficacy/

113. (empower* or self efficacy or locus of control or overconfidence).ti,ab,kw.

114. 112 or 113

115. personal autonomy/

116. (self determin* or self determination or free will or perceived autonomy or perception of autonomy or personal autonomy).ti,ab,kw.

117. 115 or 116

118. 104 or 105 or 108 or 111 or 114 or 117

119. 71 and (74 or 90 or 101 or 118)

120. Quality of Life/

121. (life quality or health-related quality of life or health related quality of life or HRQOL).ti,ab,kw.

122. 120 or 121

123. (randomized controlled trial or controlled clinical trial or evaluation studies).pt or random allocation/

124. 68 and 119 and 122 and 123

**II. Grey literature information sources**

1. Grey literature databases: Conference Proceedings Citation Index; OAIster; HSRProj; Health Services Research Projects in Progress; Trip medical database; Index to Theses; ZETOC; Archaeology data service; Grey literature report; Grey literature network; Open Grey; and EThOS
2. Trial registries: International Standard Randomized Controlled Trial Number register (ISRCTN); WHO and EU clinical trials register

(cf. Gray literature data from International Clinical Trials Registry Platform (ICTRP) and ClinicalTrials. Gov were retrieved from the Cochrane central register of trials.)
